# Impact of robotic total hip and knee arthroplasty on resident and fellow training in orthopedic surgery

**DOI:** 10.1007/s11701-025-02642-5

**Published:** 2025-08-08

**Authors:** Shujaa T. Khan, Benjamin E. Jevnikar, Ahmed K. Emara, Peter G. Delaney, Khaled A. Elmenawi, Peter A. Surace, Nicolas S. Piuzzi, Matthew Deren

**Affiliations:** Department of Orthopedic Surgery, Cleveland Clinic Foundation, Orthopedic and Rheumatology Institute, 9500 Euclid Ave, A41, Cleveland, OH 44195 USA

**Keywords:** robot, arthroplasty, replacement, education, training, resident

## Abstract

Robotic-assisted total joint arthroplasty (RA-TJA) has experienced rapid growth in adoption and is reshaping orthopedic surgical practice. Despite offering potential for improved precision, reproducibility, and intraoperative feedback, its integration into orthopedic training has raised questions about educational equity and quality, manual skill development, and trainee autonomy. This review examines the educational implications of RA-TJA on orthopedic residency and fellowship training. The goal is to synthesize existing literature on RA-TJA impact on technical skill acquisition, surgical exposure, operative autonomy, and cognitive engagement, with attention to global disparities and the role of simulation in modern training models. A narrative literature review was conducted, focusing on empirical studies, trainee surveys, and expert opinion related to robotic systems in arthroplasty education. Specific attention was given to competency-based medical education (CBME) frameworks, intraoperative performance metrics, simulation-based learning, and institutional variability. Robotic systems facilitate early technical proficiency by offering structured intraoperative workflows and objective metrics, aligning well with CBME. These platforms enhance resident involvement in preoperative planning and spatial understanding but may inadvertently reduce manual versatility and intraoperative decision-making without complementary training in conventional techniques. Trainee autonomy varies significantly by institution, and access to RA-TJA during post-graduate medical education remains inconsistent. Simulation technologies, particularly virtual reality, represent scalable solutions to bridge training gaps but require standardized integration, assessment models, and certification pathways. RA-TJA presents both opportunities and challenges for orthopedic education. Its successful implementation requires deliberate curricular design, hybrid exposure to both robotic and manual techniques, and equitable access to simulation-based resources. Moving forward, training standardization, integration of performance-based progression metrics, and global cooperation will be essential to preparing orthopedic surgeons for the evolving digital surgical landscape.

## Introduction

The increased adoption of robotic-assisted total joint arthroplasty (RA-TJA) marks a significant shift in orthopedic care, offering enhanced precision, reproducibility, and real-time intraoperative feedback compared to conventional techniques, though their benefits remain the subject of ongoing debate. Despite this, integration of robotic assistance has accelerated rapidly; from 2015 to 2020, robotic-assisted total knee arthroplasty (RA-TKA) procedures increased over 600%, and by 2030, robotic systems are projected to account for nearly half of all knee arthroplasty procedures in the United States [1–4].

As robotic systems become increasingly prevalent in clinical practice, their impact on orthopedic education has come under scrutiny. These platforms offer structured intraoperative guidance, objective performance metrics, and robust planning tools that align with competency-based medical education (CBME) models [5]. However, training integration has been inconsistent, raising important questions about educational standardization, operative autonomy, manual skill development, and equitable access. Surgical educators must determine how to harness robotic capabilities while preserving core principles of surgical training.

This narrative review was conducted using a targeted literature search of PubMed, MEDLINE, and Google Scholar to identify peer-reviewed articles, surveys, and expert consensus statements related to RA-TJA in surgical education, with particular attention to how these technologies shape technical skill acquisition, operative autonomy, surgical exposure, and cognitive engagement. Specifically, we examine: (1) how structured workflows and real-time intraoperative feedback support early proficiency but may constrain manual versatility without complementary conventional manual arthroplasty training; (2) institutional variability in robotic assistance access and its impact on case mix, procedural breadth, and exposure to revision surgery; (3) the potential of system-derived metrics to advance competency-based medical education (CBME) frameworks by enabling performance-based progression; (4) the upstream shift in decision-making introduced by robotics, which redefines how trainees engage with preoperative planning, alignment, and soft tissue management; and (5) global disparities in access to robotic education, underscoring the need for standardized curricula and simulation-based training to ensure equitable learning across diverse healthcare environments.

## Technical skill acquisition

RA-TJA platforms have introduced a structured, data-rich environment for the acquisition of surgical skills in orthopedic training. These systems enable real-time intraoperative feedback on technical parameters including implant positioning, bony resection depth, alignment, and gap balancing, facilitating deliberate practice and immediate error correction. In contrast to conventional manual techniques, which often rely on subjective intraoperative cues and experiential learning, robotic systems provide an objective scaffold for technical development in early learners [6–8].

The pedagogical value of robotics can be contextualized through the framework of Fitts and Posner’s three-stage model of motor learning. During the cognitive and associative phases, where performance is variable and feedback-dependent, robotic platforms serve as a high-fidelity interface that guides performance toward reproducible, biomechanically sound outcomes. In addition, some robotic platforms incorporate haptic boundaries and guided planning workflows, reducing the likelihood of errors and allowing novices to perform complex tasks within predefined safety parameters. This structured autonomy may accelerate skill acquisition without compromising patient safety [6].

Empirical data support this theoretical foundation. Shapira et al. demonstrated that evaluating estimated implant version and inclination by fellows using RA-THA demonstrated many cups may have been placed outside the Lewinnek safe zones if not for robotic or navigation systems providing feedback to understand their own inaccuracies and highlighted its utility in reinforcing spatial accuracy [7]. In a randomized controlled trial, Saad et al. showed that trainees who completed robotic software-based modules for total knee arthroplasty exhibited significantly improved understanding of mechanical alignment, joint line restoration, and ligament balancing, compared to peers trained through conventional didactics [8]. Further, Kayani et al. conducted a prospective cohort study analyzing the early adoption of RA-TKA and demonstrated that, although operative times and surgical team anxiety improved after approximately seven cases, the accuracy of implant positioning and limb alignment was consistently superior to manual techniques from the outset [9]. These findings suggest that robotic platforms can provide a stable and reliable operative environment even during the learning phase, enabling trainees to participate in technically accurate procedures early in their experience without sacrificing educational quality. Additionally, with more studies demonstrating ideal implant position for specific cases being different from historic targets of “safe zones”, determining these positions and achieving them accurately can be improved with robotic assistance.

Finally, robotic systems offer a quantifiable means of assessing technical competence, an increasingly important element of CBME. Parameters such as planned-versus-achieved implant position, bone resection error, and ligament balance symmetry can serve as objective metrics of proficiency, enabling data-driven competency evaluations. This paradigm aligns with emerging calls for outcomes-based progression models in surgical education, where advancement is contingent upon demonstrated skill, rather than a pragmatic case count approach [9–11].

## Case volume and surgical exposure

Case volume remains a cornerstone of surgical education, directly influencing the development of operative confidence, technical proficiency, and decision-making autonomy. In the context of RA-THA and RA-TKA, the proliferation of robotic platforms across academic and private practice settings introduces complex implications for both the quantity and diversity of cases encountered during residency and fellowship training.

### Impact on case mix and procedural breadth

The introduction of RA-TJA may alter the proportion and complexity of arthroplasty cases. Kolessar et al. observed a 28% increase in TKA volume after the implementation of RA-TKA, largely due to expanded indications and increased confidence in performing unicompartmental knee arthroplasty (UKA) [12]. Though UKA is historically underrepresented in residency case logs, increased RA-TKA utilization may enable increased trainee exposure at equipped centers. Moreover, robotic platforms now enable technically demanding procedures, such as UKA-to-TKA conversions and revision TKA, with greater accuracy, further enriching resident and fellow learning. In a recent report, Piuzzi et al. demonstrated the use of robotic-assisted conversion of UKA to TKA, emphasizing the advantages of improved bone preservation, decreased reliance on augments, and enhanced alignment precision [13]. This not only reinforces the versatility of robotic assistance, but also exposes trainees to revision-specific skills such as dynamic soft tissue balancing and templated resection planning. In this context, robotic arthroplasty may serve not only as a teaching tool for primary arthroplasty principles, but as a gateway to advanced reconstructive concepts.

Exposure alone is insufficient to guarantee competence using robotic assistance. Educational value depends on the extent to which they are deliberately integrated into structured training. In a retrospective analysis, Deckey et al. demonstrated that resident participation in RA-TKA did not prolong operative time, indicating that robotic workflows can support intraoperative teaching without compromising efficiency [6]. Additionally, certain robotic platforms may allow enhanced visualization for the surgeon while the trainee operates and can include haptic bounds for saw and reamer attachments, ultimately allowing graduated autonomy in a way that is safer than previously seen with manual arthroplasty training.

Nonetheless, survey-based studies continue to highlight wide variability in trainee autonomy and involvement, likely reflecting institutional differences in mentorship models, workflow design, and access to robotic systems [14]. To realize the full pedagogical potential of robotic technology, orthopedic training programs must move beyond passive exposure and adopt curricula that incorporate longitudinal simulation-based learning, graduated intraoperative responsibility, and concurrent instruction in conventional manual arthroplasty techniques.

### Surgical volume and competency milestones

Historically, post-graduate surgical education has long emphasized procedural volume as a pragmatic proxy indicator for trainee competence. However, RA-TJA challenges this paradigm by offering reproducible workflows that may accelerate technical proficiency at lower case counts. In a study of RA-TKA implementation, Kayani et al. found no learning curve for implant positioning accuracy, even in early cases, suggesting that robotic guidance can facilitate rapid acquisition of core technical skills [9]. From a training standpoint, this implies that residents may achieve proficiency with fewer repetitions when supported by robotic feedback.

In this context, metrics generated by robotic systems including bone resection error, implant alignment deviation, and soft tissue balance may serve not only as learning tools, but as competency benchmarks. These intraoperative metrics might be used to quantify surgical competence more precisely than case logs alone, aligning with growing momentum toward CBME models advocating for progression based on performance rather than experience thresholds [10, 11]. As training models shift toward outcomes-based progression, system-derived metrics may support simulation-based credentialing, milestone tracking, and standardized benchmarks across institutions and the abandonment of volume as a pragmatic alternative.

Finally, while RA-TJA case volume may remain limited at some institutions, simulation technologies like virtual reality (VR) offer a scalable adjunct to technical skill development. VR modules for THA and TKA have been shown to improve visuospatial precision, procedural sequencing, and surgical efficiency, with demonstrated skill transfer to cadaveric and live surgical settings [15, 16]. Though not yet integrated with robotic workflows, these platforms may help bridge early proficiency gaps and serve as preparatory tools, particularly in low-volume settings or for early-stage post-graduate trainees.

### International considerations and the need for standardization

As RA-TJA is increasingly adopted globally, simulation-based education including VR, synthetic models, and digital planning platforms has emerged as an integral component of international training efforts. Rather than serving solely as technical rehearsal tools, these platforms are increasingly used to deliver structured, reproducible curricula that align with competency-based educational models. In the context of global training, VR offers the potential to standardize instruction across regions with disparate clinical volumes or robotic assistance access, supporting skill development even in resource-limited environments [15–17]. These platforms support the broader shift toward competency-based curricula, which favor structured feedback, deliberate practice, and proficiency-based progression over traditional volume-based milestones [18, 19].

However, access to simulation-based training remains highly inequitable. In low- and middle-income countries (LMICs), widespread adoption is impaired primarily by a shortage of trained surgeons and faculty, as sub-Saharan Africa has less than 1% of the number of surgeons in the United States, despite having a population that is three times as large, owing to a low number of medical school graduates, inadequate training, the inability to retain staff in remote regions, as well as poor salaries and working conditions [20]. Additionally, limited infrastructure, supply chain, and financial constraints further limit uptake [21]. Surgical education in these settings continues to rely heavily on didactics and opportunistic intraoperative learning, often without structured simulation curricula or assessment frameworks [21]. Even in higher-resource settings, variability exists in how robotic training is delivered and evaluated, further underscoring the need for consistent standards [22].

Several international collaborations have emerged to address these disparities. Telesimulation, low-cost VR modules, and traveling faculty partnerships have shown early promise in providing scalable access to structured training [5, 21]. Institutions in high-income countries have partnered with LMICs to co-develop context-specific curricula, train local simulation instructors, and establish affordable, sustainable simulation centers [21]. Bidirectional partnerships between LMIC hospitals and high-income country academic institutions have further enabled global academic orthopaedic collaborations, and may be a model for further sustainable capacity building [23].

Best practices in simulation-based surgical education emphasize integration into formal curricula with clearly defined objectives, structured feedback, and validated assessment tools such as the Objective Structured Assessment of Technical Skills [24]. Simulation must complement, not replace, traditional operative experience, and robotic training should be paired with manual arthroplasty techniques to ensure comprehensive skill development and adaptability across different practice environments [19, 24].

To date, there is no universally accepted standard for robotic surgery training in orthopedic education. The heterogeneity in training models across institutions and nations reflects a broader need for globally coordinated efforts to define proficiency benchmarks, standardize curricula, and promote equitable access to technology-enhanced education. As RA-TJA expands worldwide, coordinating training standards will be essential to ensure quality, safety, and preparedness across diverse healthcare systems.

## Operative autonomy and decision-making

RA-TJA alters the landscape of operative autonomy and surgical decision-making in profound and complex ways. While traditional autonomy has been defined by manual execution of technical steps, robotic systems reframe autonomy around case planning, software navigation, and intraoperative system control. This shift carries both opportunities and challenges for surgical trainees.

### Operative autonomy in the robotic era

The integration of robotic systems into TJA has created a complex and often contradictory landscape for operative autonomy among orthopedic trainees. While robotic platforms offer potential educational benefits, including structured workflows and real-time feedback, their impact on resident autonomy remains variable. Survey data consistently demonstrate that although exposure to robotic-assisted procedures is becoming increasingly common, meaningful hands-on experience and graduated autonomy are less prevalent. In a national survey of orthopedic trainees, only 44.4% of those with RA-TJA training reported being granted increasing autonomy in robotic cases [14]. Moreover, the majority of residents expressed discomfort with independently using robotic systems, despite a widespread belief in their educational value (71.4%) and their expected permanence in clinical practice (90.7%) [14].

**Fig. 1:**
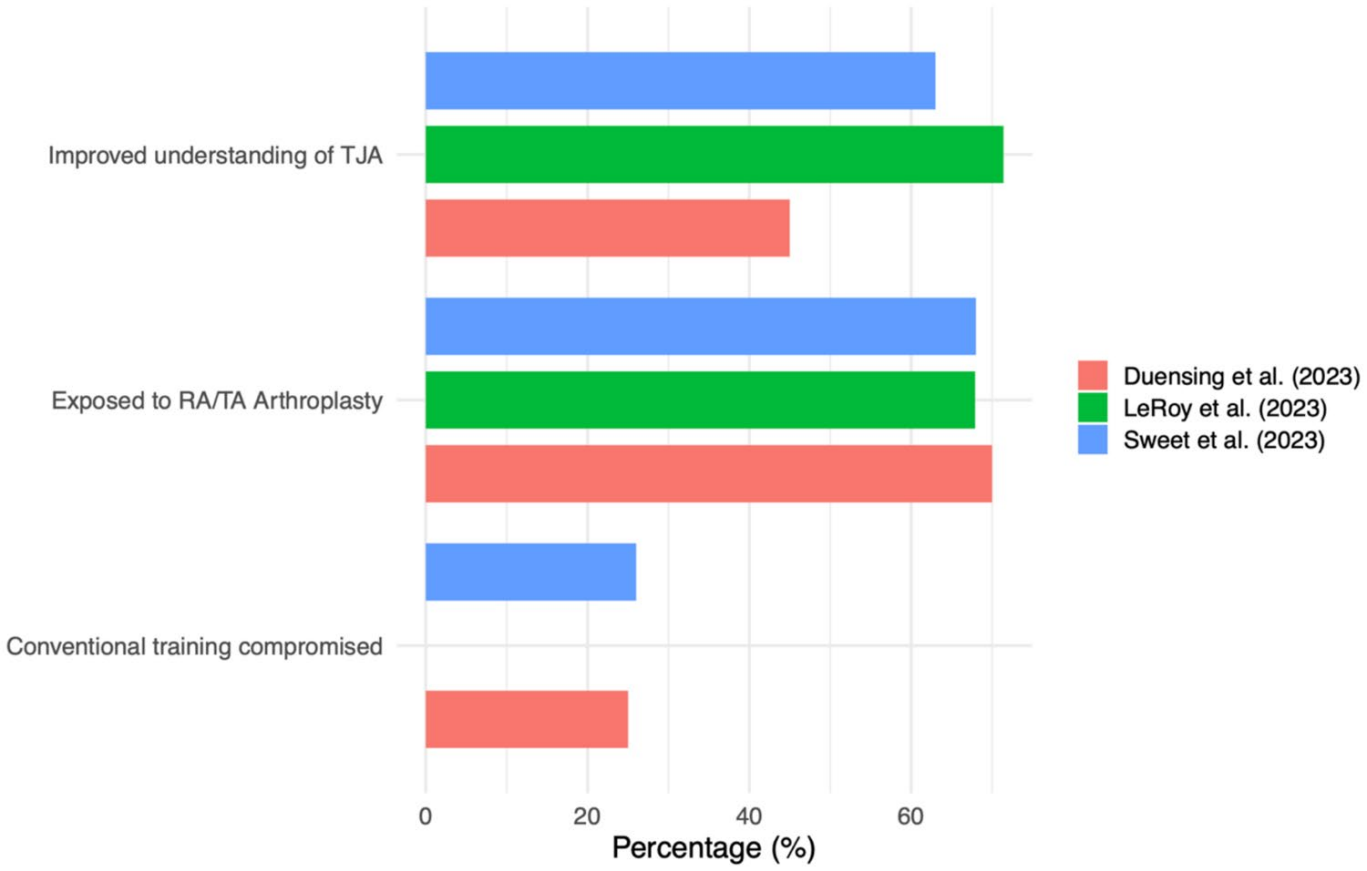
Orthopedic resident perceptions of robotic- and technology-assisted total joint arthroplasty (RA/TA-TJA)

Importantly, greater exposure to RA-TJA has been associated with both benefits and trade-offs. While trainees with higher case volumes report improved understanding of procedure mechanics and templated workflows, this exposure may come at the cost of decreased comfort with traditional instrumentation. Duensing et al. found that 25% of residents felt their training in conventional techniques was negatively impacted by robotic adoption [5], and Sweet et al. similarly reported 26% of trainees worried their non-robotic arthroplasty training was insufficient due to overemphasis on robotic-assisted cases (Fig. 1) [25]. These findings highlight a pedagogical tension: robotic platforms can enhance cognitive and visual-spatial learning, but may inadvertently erode manual arthroplasty proficiency and sacrifice rich manual operative exposures if not thoughtfully balanced with conventional experience.

Nonetheless, the effect of robotics on resident autonomy is highly dependent on institutional culture, attending surgeon preferences, and the presence of structured training pathways. In programs where robotic systems are integrated without parallel investment in faculty development or curriculum design, resident involvement may remain superficial. Conversely, institutions that promote progressive entrustment, in order to encourage resident participation in preoperative planning, intraoperative system control and interpretation of robotic feedback, can leverage these platforms to meaningfully support graduated autonomy.

### Cognitive engagement and preoperative planning

RA-TJA introduces a digitally structured workflow that enhances the cognitive engagement of orthopedic residents by shifting critical surgical decisions to the preoperative phase. Unlike conventional arthroplasty, where intraoperative decisions dominate, robotic systems demand early input on implant sizing, component alignment, and bone resection parameters. These tasks require deliberate planning and an understanding of biomechanical targets. Residents who participate in this planning process benefit from an enriched educational experience. Real-time feedback, three-dimensional modeling, and stepwise execution reinforce core concepts in alignment strategy, bony morphology, and gap balancing. These elements promote active learning and may improve conceptual understanding of total joint biomechanics. In survey data, 45% of residents reported improved understanding of arthroplasty procedures due to robotic exposure, and over 70% believed robotic systems enhanced their education (Fig. 1) [5, 14].

Yet, concerns have been raised regarding cognitive offloading in early trainees. Although RA-TJA enhances planning literacy and supports early cognitive engagement, its reliance on preoperative templating, intraoperative guidance, and automated execution of bone cuts introduces the risk of premature cognitive offloading in trainees by reducing the need for active intraoperative decision-making, particularly in early learners not involved in the pre-operative planning stage. Thus, RA-TJA may stunt the development of independent surgical judgment and technical adaptability required in later stages. To mitigate this risk, future training paradigms should incorporate deliberate cognitive checkpoints, such as guided templating exercises, intraoperative plan validation, and real-time reasoning prompts, to ensure that trainees remain actively engaged in critical decision-making processes throughout the case, when not involved pre-operatively in templating and planning [6, 26, 27].

To realize the cognitive benefits of RA-TJA, residency programs must integrate trainees into all phases of the robotic workflow, from preoperative plan creation to intraoperative troubleshooting. Didactic sessions, case-based planning modules, and structured feedback on templating decisions can help reinforce surgical reasoning. Additionally, curricular balance must be maintained to ensure that conceptual learning via robotic systems is paralleled by continued exposure to conventional instrumentation and unassisted decision-making.

## Complication profile in robotic and manual techniques

Recent literature demonstrates that RA-TJA and manual techniques have broadly similar overall complication profiles, with some procedure- and technology-specific differences. After residency training, complication rates are not increased by trainee involvement, and the presence of structured residency programs may even be associated with reduced complication rates in teaching hospitals [28–30].

For THA, large matched cohort studies and meta-analyses show that RA-THA is associated with lower odds of 90-day revisits and readmissions for dislocation and surgical site infection compared to manual THA, with no significant difference in overall emergency department visits or all-cause complication rates [31, 32]. Some registry data suggest minor increases in certain complications (e.g., myocardial infarction, wound dehiscence) with RA-THA, but these differences are small and not consistently observed across studies [33]. Importantly, both techniques demonstrate similar short-term and two-year revision-free survival [31–34].

RA-TKA is associated with lower rates of 90-day hospital revisits, readmissions for joint stiffness, and certain perioperative complications (e.g., intraoperative fracture, respiratory complications, postoperative anemia, thromboembolic events) compared to manual TKA [35, 36]. However, some single-center registry data indicate a higher rate of wound complications and periprosthetic joint infection revisions in cemented RA-TKA, while cementless RA-TKA may reduce postoperative pain compared to manual techniques [37]. Overall, both approaches have similar revision rates and comparable 1-year patient-reported outcomes [38, 39].

Interestingly, resident involvement in TJA does not increase orthopedic complication or revision rates for either THA or TKA, though operative times may be slightly longer and non-orthopedic complications marginally higher in resident-performed cases [28]. The introduction of ACGME-accredited residency programs has been associated with a reduction in complication rates for lower extremity TJA at teaching hospitals, without affecting readmission rates [28, 30].

In summary, robotic-assisted and manual techniques in TJA after residency training yield similar overall complication profiles, with robotic assistance conferring modest reductions in some short-term complications and readmissions. Resident involvement does not adversely affect complication rates, supporting the safety of both approaches in the post-training setting.

## Fellowship exposure and long-term practice patterns

Fellowship training plays a pivotal role in shaping the operative habits, technology preferences, and clinical decision-making frameworks of arthroplasty surgeons. This formative period often serves as the primary window in which surgeons are exposed to specific surgical approaches, implant systems, and enabling technologies such as robotic-assisted workflows. While the long-term impact of fellowship exposure is difficult to quantify, numerous reports and expert consensus suggest that what is learned during fellowship frequently becomes the foundation of future practice [40, 41].

RA-TJA offers a particularly illustrative example. Adoption of robotic technology varies substantially across institutions, and thus, exposure of orthopaedic fellows to specific platforms is often dictated by site availability rather than by standardized curriculum. In this context, the type of robot encountered during training may directly influence future adoption, with many early-career surgeons continuing to use the same systems and vendors they were trained on. Conversely, limited exposure during fellowship may hinder a surgeon’s comfort with or willingness to incorporate robotic workflows later in independent practice. This underscores the importance of structured, vendor-neutral training that emphasizes adaptability and cross-platform competency rather than platform-specific proficiency.

Importantly, recent survey data highlight that early-career surgeons are increasingly embracing robotic technology. In the most recent American Association of Hip and Knee Surgeons (AAHKS) membership poll, 53% of respondents reported using robotics in TKA [42], a sharp rise from 21% in 2018 [43], while 23% reported using robotic assistance in THA [42]. Overall, these trends suggest a generational shift aligned with evolving fellowship experiences and broader technological adoption in arthroplasty.

As robotic systems and other advanced technologies become more prevalent, fellowship programs must respond by expanding structured exposure, offering simulation-based training, and providing longitudinal involvement in planning and execution. Exposure should not be incidental, but rather a deliberate component of the educational mission. Preparing fellows to evaluate and adapt to new technologies, not simply to operate within the bounds of one system, will be key to building future-ready, autonomous surgeons in a rapidly advancing field.

## Future implications

As RA-TJA continues to evolve, its integration into orthopedic training programs will increasingly define the competencies of the next generation of surgeons. The current evidence suggests that robotic platforms enhance cognitive engagement in the preoperative period, facilitate early technical proficiency, and offer a rich, data-driven environment for feedback and assessment. However, these benefits are tempered by concerns regarding diminished hands-on autonomy, uneven institutional adoption, and the potential hindrance of manual surgical skills. Moving forward, the challenge will not be whether robotics belongs in surgical education, but how it is implemented, balanced, and evaluated across diverse training landscapes.

One critical implication lies in curricular design. Robotic workflows demand a recalibration of training models to ensure cognitive and technical development proceed in parallel. CBME frameworks offer a promising structure, emphasizing milestone progression and proficiency-based assessment. Robotic platforms are uniquely positioned to support these models, as they can generate objective intraoperative metrics including implant accuracy, resection precision, and gap balance symmetry, which may serve as quantifiable metrics for resident competency. To operationalize these curricular shifts, formalized credentialing pathways should be developed. Trainees may benefit from standardized proficiency assessments, focused on robotic system navigation, error recognition, and intraoperative decision-making.

Simulation will also play an increasingly vital role, particularly in equalizing access and reinforcing skill acquisition in low-volume or resource-constrained settings. Fully immersive VR platforms and telesimulation offer scalable training solutions that can supplement limited robotic case volume. However, these tools must be deliberately integrated into educational ecosystems, paired with feedback mechanisms, and contextualized within real-world clinical decision-making. Simulation could be used not only as a technical rehearsal but also as a cognitive primer for surgical planning, templating, and intraoperative adjustment. In addition, cross-platform simulation modules, designed independent of specific robotic vendors, could promote flexible, vendor-neutral learning and reduce institutional bias in technology exposure.

Institutionally, training programs must be proactive in avoiding overreliance on robotics to the exclusion of conventional techniques. A hybrid model, leveraging robotics for its cognitive and precision advantages while maintaining manual exposure for tactile and adaptive skill development, is essential. Graduates must be competent across the spectrum, equally capable of performing a RA-TKA and converting to a manual approach when indicated. This balance requires intentional case distribution, faculty development, and rotation structuring to ensure residents are not passive observers but active participants in both planning and execution of RA-TJA and conventional manual approaches.

Finally, orthopaedics must move toward standardization. Currently, robotic training varies widely by institution, with no unified curriculum or certification pathway. Orthopedic governing bodies such as the Accreditation Council for Graduate Medical Education (ACGME), American Academy of Orthopaedic Surgeons (AAOS), and American Association of Hip and Knee Surgeons (AAHKS) should collaborate to establish minimum case exposure, simulation training hours, and platform competencies required for graduation. These benchmarks would help equalize access and ensure training consistency across institutions. International collaborations, particularly those aimed at expanding access to simulation and robotic education in LMICs, will be critical to closing the global training gap and ensuring that advancements in surgical technology do not exacerbate disparities in surgical education. Additionally, training programs must continue to adapt as robotic platforms advance and further features become available.

In summary, RA-TJA offers a powerful opportunity to modernize orthopedic training, but its implementation must be guided by intentional design, curricular balance, and a commitment to preserving the foundational principles of surgical competence. As the field advances, the question for educators is not simply how to teach trainees to use the robot, but how to ensure they think, adapt, and operate as fully independent surgeons in an increasingly digital operating room.

## Limitations

This review is subject to several limitations inherent to its narrative methodology. First, although a targeted literature search was conducted, this review does not represent a systematic or scoping review and may have omitted relevant studies, particularly those published outside of indexed databases or in non-English languages. Second, the available literature on RA-TJA in residency and fellowship education remains limited, with much of the current evidence drawn from single-institution experiences, survey-based data, expert opinion, or observational studies. As such, generalizability across diverse training environments is constrained. Third, heterogeneity among robotic platforms, institutional adoption models, and training curricula poses challenges to drawing definitive conclusions about the impact of RA-TJA on trainee outcomes. Differences in surgical volume, case mix, faculty engagement, and access to simulation technologies all influence the educational experience, yet are often inconsistently reported or controlled for in the literature. Finally, this review is limited by the rapidly evolving nature of robotic technology and educational models; findings and recommendations may require re-evaluation as newer systems and training frameworks are developed.

Despite these limitations, this review aims to synthesize the best available evidence and provide a conceptual framework for understanding how robotic arthroplasty may influence orthopedic education. Future work should prioritize multicenter, prospective studies, competency-based outcome tracking, and the development of standardized curricula to better inform best practices in this emerging domain.

## Conclusion

RA-TJA is transforming orthopedic education, introducing a digitally structured environment that enhances technical precision, cognitive engagement, and feedback-driven learning. These platforms support early proficiency and align well with competency-based educational models, but their implementation also presents notable challenges. Disparities in case exposure, diminished manual autonomy, and uneven curricular integration highlight the importance of thoughtful educational design. As training programs seek to modernize surgical education, a balanced approach that integrates robotic and conventional techniques, emphasizes active participation across all stages of operative planning and exectuion, and leverages data to inform competency progression is imperative. The future of orthopedic training lies not in choosing between tradition and technology, but in integrating both to prepare trainees for a rapidly evolving surgical landscape. Deliberate curriculum development, equitable access to simulation, and global standardization will be key to ensuring that the next generation of surgeons is not only adept with robotic systems but also grounded in the foundational principles of surgical judgment, adaptability, and autonomy.

## Conflict of interests

Dr. Shujaa T. Khan, Mr. Benjamin E. Jevnikar, Dr. Ahmed Emara, Dr. Peter G. Delaney, and Dr. Khaled A. Elmenawi have no conflicts of interest. Dr. Peter A. Surace reports Other Professional Activities from Stryker. Dr. Nicolas S. Piuzzi reports research support from Osteal Therapeutics, Zimmer-Biomet, Peptilogics, RegenLab, and Signature Orthopaedics; consulting for Pacira and Stryker; and board/editorial roles with AAHKS, ISCT, and The Journal of Bone and Joint Surgery. Dr. Matthew E. Deren reports honoraria from Brasseler, stock in Romtech, and serves on the editorial board of The Journal of Knee Surgery.

## Data Availability

No datasets were generated or analysed during the current study.
